# Targeting Features of the Metabolic Syndrome Through Sympatholytic Effects of SGLT2 Inhibition

**DOI:** 10.1007/s11906-022-01170-z

**Published:** 2022-03-02

**Authors:** Lakshini Y. Herat, Jennifer Matthews, Omar Azzam, Markus P. Schlaich, Vance B. Matthews

**Affiliations:** 1 Dobney Hypertension Centre, School of Biomedical Science - Royal Perth Hospital Unit, University of Western Australia, MRF Building, Level 3, Rear 50 Murray St, Perth, WA 6000 Australia; 2 Royal Perth Hospital Research Foundation, Royal Perth Hospital, Perth, WA Australia; 3 Department of Medicine, Royal Perth Hospital, Perth, WA Australia; 4 Dobney Hypertension Centre, School of Medicine, Royal Perth Hospital Unit, University of Western Australia, Perth, Australia; 5 Department of Cardiology and Department of Nephrology, Royal Perth Hospital, Perth, Australia

**Keywords:** Hypertension, Metabolic syndrome, SGLT2 inhibition, Sympatho-inhibition, Sympathetic nervous system, Cardio-renal protection

## Abstract

**Purpose of Review:**

The moderate glucose-lowering effect of sodium glucose co-transporter 2 (SGLT2) inhibitors is unlikely to explain SGLT2 inhibitor-mediated beneficial outcomes, and unravelling the underlying mechanisms is a high priority in the research community. Given the dominant pathophysiologic role of the sympathetic nervous system activation in conditions such as hypertension and perturbed glucose homeostasis, it is pertinent to postulate that SGLT2 inhibitors may exert their beneficial effects at least in part via sympathetic inhibition.

**Recent Findings:**

SGLT2 inhibitors have shown enormous potential to improve cardiovascular outcomes in patients with type 2 diabetes, and their therapeutic potential is currently being investigated in a range of associated comorbidities such as heart failure and chronic kidney disease. Indeed, recent experimental data in relevant animal models highlight a bidirectional interaction between sympathetic nervous system activation and SGLT2 expression, and this facilitates several of the features associated with SGLT2 inhibition observed in clinical trials including improved glucose metabolism, weight loss, increased diuresis, and lowering of blood pressure.

**Summary:**

Currently available data highlight the various levels of interaction between the sympathetic nervous system and SGLT2 expression and explores the potential for SGLT2 inhibition as a therapeutic strategy in conditions commonly characterised by sympathetic activation.

## Introduction

The sympathetic nervous system (SNS) is a crucial player in circulatory and metabolic control [1, 2]. Increased sympathetic outflow to the heart results in increased cardiac output mediated by an increase in heart rate and stroke volume. Increased sympathetic outflow directed toward the kidneys causes sodium retention, increased renin release from the juxtaglomerular apparatus and alterations in renal blood flow. Furthermore, a systemic peripheral vasoconstrictor effect ensues sympathetic activation. It is obvious that these effects contribute substantially to blood pressure (BP) elevations, both acutely and in the long term, particularly if occurring simultaneously and/or if sympathetic activation is sustained over a longer period of time.

It is generally less well appreciated that SNS activation also has profound metabolic effects [1, 2]. An acute rise in sympathetic activity results in increased lipolysis and increased levels of fatty acids in plasma, increased gluconeogenesis by the liver to provide substrate for the brain and moderate inhibition of insulin release by the pancreas to conserve glucose and to shift fuel metabolism of muscle in the direction of fatty acid oxidation, inflammation and others [3, 4]. If sympathetic activity is raised chronically due to an unfavourable lifestyle, however, the physiologic responses may take an unfavourable direction, including the development of increased fasting glucose levels and insulin resistance, as well as elevated BP and hypertension, both of which are critical features of the metabolic syndrome (MetS) [5–7].

The metabolic syndrome which is characterized by the concurrent occurrence of a cluster of metabolic abnormalities such as central (abdominal) obesity, elevated fasting glucose, dyslipidaemia (elevated triglycerides and/or low high-density lipoproteins (HDL)-cholesterol) and elevated BP is directly associated with an increased risk of cardiovascular (CV) disease, type 2 diabetes and all-cause mortality [8]. Furthermore, the MetS is highly prevalent in patients with various forms of hypertension [9, 10]. In hypertensive subjects, the MetS amplifies CV risk associated with hypertension, independent of the effect of traditional CV risk factors such as lack of physical activity, smoking and the presence of susceptibility genes [9]. A wealth of evidence suggests a bidirectional relationship between insulin resistance/hyperinsulinemia and SNS activation [11]. As a result of heightened SNS activity, a cascade of events is triggered where the kidneys increase sodium reabsorption and release renin, the heart increases cardiac output and arteries respond with vasoconstriction, all contributing to a rise in BP and if sustained for prolonged periods of time establishes hypertension [12].

As shown by Grassi et al., sympathetic nerve activity was significantly greater in subjects with MetS both with and without hypertension than in control subjects and correlated directly and significantly with the HOMA (homeostasis model assessment) index, a variable reflecting insulin resistance. These findings confirmed the notion that hypertension in the MetS initiates sympathetic activation to a greater magnitude than when hypertension is excluded [13]. Even in young subjects with mild obesity, there was evidence of substantial sympathetic activation when compared to age-matched lean control subjects [14].

In addition, Mahfoud et al. reported that reduction of sympathetic activity by renal denervation in patients with resistant hypertension substantially improved glucose metabolism and insulin sensitivity, in addition to markedly reducing BP [15]. These findings significantly added to the concept that sympathetic activation underlies the origin of both hypertension and MetS. Therefore, in this review, we discuss the beneficial sympatholytic effects that the novel drug class, sodium glucose co-transporter 2 (SGLT2) inhibitors have on hypertension in the MetS.

## SGLT2 Inhibitors: Glucose Lowering and Beyond

The renal mechanisms and the essential involvement of the kidneys in glucose metabolism are well documented. Typically, ∼ 180 g/day of glucose is filtered by the glomeruli of the kidneys, and almost all of this is subsequently reabsorbed in the renal proximal convoluted tubule. This reabsorption is predominantly (~ 90–95%) affected by the high-capacity, low-affinity glucose co-transporter known as SGLT2 which is expressed in the S1 segment of the renal proximal tubular epithelial cells [16]. In patients with diabetes mellitus, SGLT2 inhibitors increase glucosuria by blocking glucose reabsorption in the renal proximal tubule, and hence lower plasma glucose levels, independent of insulin stimulation [17]. When compared to other glucose-lowering medications, the glycaemic efficacy of SGLT2 inhibitors is considered to be relatively modest with 0.4 to 1.1% reduction in haemoglobin A1c (HbA1c) levels [18, 19]. According to regulatory approvals, the initiation of SGLT2 inhibitors is currently not recommended to individuals presenting with an estimated glomerular filtration (eGFR) of < 45 mL/min/1.73 m^2^.

In patients with diabetes mellitus, treatment with SGLT2 inhibitors has been shown to be associated with cardiometabolic benefits such as weight loss, [20, 21] BP reduction [22] and improvements in lipid profiles [23, 24]. In studies involving ApoE − / − mice, three of the currently available SGLT2 inhibitors including empagliflozin (EMPA), dapagliflozin (DAPA) and canagliflozin (CANA) all significantly reduced triglyceride levels, and EMPA also raised HDL levels [25, 26••, 27].

Additionally, studies have shown that inflammation promotes the development of atherosclerosis, by increasing endothelial dysfunction, lipid oxidation and plaque destabilisation/rupture, another important pathophysiologic mechanism that ideally should be targeted therapeutically [26••]. Indeed, three of the most well-known SGLT2 inhibitors (EMPA, DAPA and CANA) have all been shown to play vital roles in decreasing inflammation in Apo E − / − mice. Empagliflozin has been shown to significantly reduce IL-1β, IL-6 and IL-10 levels [26••], while DAPA significantly reduces NLRP3, IL-1β and IL-18 [27], and CANA significantly reduces the adhesion molecule, VCAM-1 and decreases MCP-1, while increasing the TIMP-1 inhibitor [25].

**Fig. 1:**
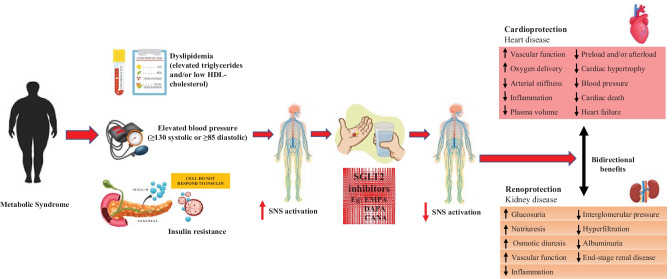
Beneficial effects of sodium glucose co-transporter 2 (SGLT2) inhibition on metabolism which are mediated by sympathoinhibition. Administration of SGLT2 inhibitors decreases obesity-induced metabolic dysfunction as evidenced by decreased activation of the sympathetic nervous system which promotes improvements in glucose homeostasis and cardiorenal protection

Furthermore, beyond and independent of glycaemic control, clinical trials using SGLT2 inhibitors have demonstrated unprecedented cardio-renal benefits such as significantly reduced CV morbidity and mortality, lower rates of hospitalized heart failure, improved renal function and reduced progression of diabetic nephropathy [20, 28, 29••, 30, 31] (Fig. 1). These interesting findings will later be discussed in detail.

## Underlying Mechanisms Pertaining To the Benefits of SGLT2 Inhibition on Hypertension

**Fig. 2:**
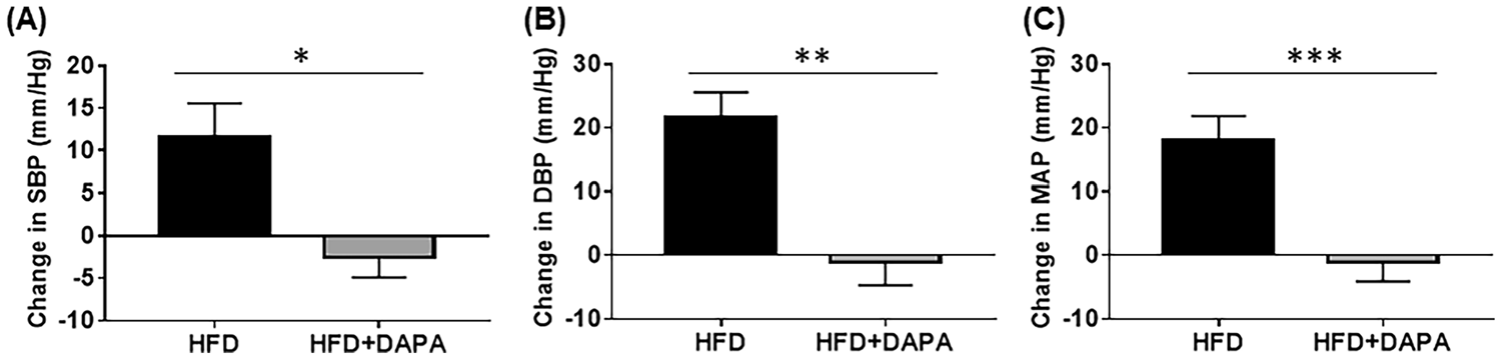
Sodium glucose co-transporter 2 inhibition with dapagliflozin (DAPA) reduces blood pressure in hypertensive mice. Effects of DAPA on **A** systolic blood pressure, **B** diastolic blood pressure and **C** mean arterial blood pressure using tail-cuff apparatus, *n* = 10–12 mice/group; *p = 0.006; **p = 0.0003; ***p = 0.007; mean ± SEM [43••]

Although the precise mechanisms of BP reduction initiated by SGLT2 inhibitors are not fully understood, a large number of randomised controlled trials in patients with type 2 diabetes have documented reductions in BP when treated with SGLT2 inhibitors [32]. Observations from various groups including ours confirmed a significant BP-lowering effect of SGLT2 inhibition in various animal models including one of neurogenic hypertension, in this instance treated with DAPA (Fig. 2). Several underlying pathophysiologic mechanisms have been proposed in current literature including osmotic diuresis, mild natriuresis, weight loss and reduced sympathetic tone [33].

A study conducted in people with normal kidney function demonstrated that the BP reduction earlier in the SGLT2 inhibitor treatment regime is potentially associated with plasma volume depletion caused by osmotic diuresis. However, the BP-lowering effect at the latter phase of this study was due to natriuresis or urinary sodium excretion [34].

Studies have observed a reduction of 2–3 kg of body weight with the treatment of SGLT inhibitors. This reduction has been associated with the increased loss of calories via urinary glucose excretion and osmotic diuresis of this drug class [35]. A meta-analysis of 25 randomised controlled trials has shown that, on average, a − 1.05 mmHg reduction of systolic BP and − 0.92 mmHg reduction of diastolic BP can be achieved per kilogram of body weight loss [36]. The SGLT2 inhibitor CANA provided clinically meaningful body-weight reductions, and each 1% reduction in body weight was associated with a 0.62-mmHg reduction in systolic BP in patients with type 2 diabetes [37].

Both natriuretic and osmotic diuresis leads to decreased extracellular volume, combined with further long-term body weight reduction, and it is thought to contribute in part to the decreased systolic BP (~ 5 mmHg) and diastolic BP (~ 2 mmHg) which is observed with all SGLT2 inhibitors [22, 38]. The anti-hyperglycaemic effects of SGLT2 inhibitors are reduced in type 2 diabetic patients with reduced GFR. However, body weight reduction, BP lowering, and heart failure protective effects are preserved in patients with chronic kidney disease and reduced eGFR (eGFR ≥ 30 ml/min/1.73m^2^) [39, 40]. This finding suggests that SGLT2 inhibition in patients with type 2 diabetes and chronic kidney disease or reduced total GFR potentially preserves lasting natriuretic and diuretic effects. From a clinical perspective, the diuretic action of SGLT2 inhibitors must be taken into consideration when prescribing this drug class, particularly in patients already on diuretics for hypertension, heart failure or chronic kidney disease. Volume depletion is a potential side effect of SGLT2 inhibition and may lead to adverse health outcomes in susceptible cohorts such as the elderly and those with impaired kidney function.

Furthermore, SGLT2 inhibition may also reduce BP by mechanisms unrelated to glucose lowering such as improved arterial stiffness [41] and endothelial dysfunction suggesting direct vascular effects [42, 43••], improved renal renin-angiotensin system activity [44] and reduced oxidative stress [45]. It is noteworthy that SGLT2 inhibitors reduce BP despite an absence of an increase in heart rate [46••, 47]. This indirectly suggests that the use of these agents may indeed be associated with a reduction in SNS activity.

## SGLT2 Inhibitor Mediated Sympathoinhibition to Improve Metabolic Control

Given the central role sympathetic overactivity plays in metabolic abnormalities such as hypertension, inhibition of the SNS is a logical and attractive therapeutic approach to treat hypertension in the MetS, and this could potentially improve the metabolic profile and reduce CV disease risk. Excessive central sympathetic activation has been shown to be reduced by lifestyle modifications such as aerobic exercise training, weight loss and stress reduction [48]. A multitude of studies suggest that pharmacological interventions with SGLT2 inhibitors may target the excessive central sympathetic activation and therefore result in concomitant metabolic benefits.

**Fig. 3:**
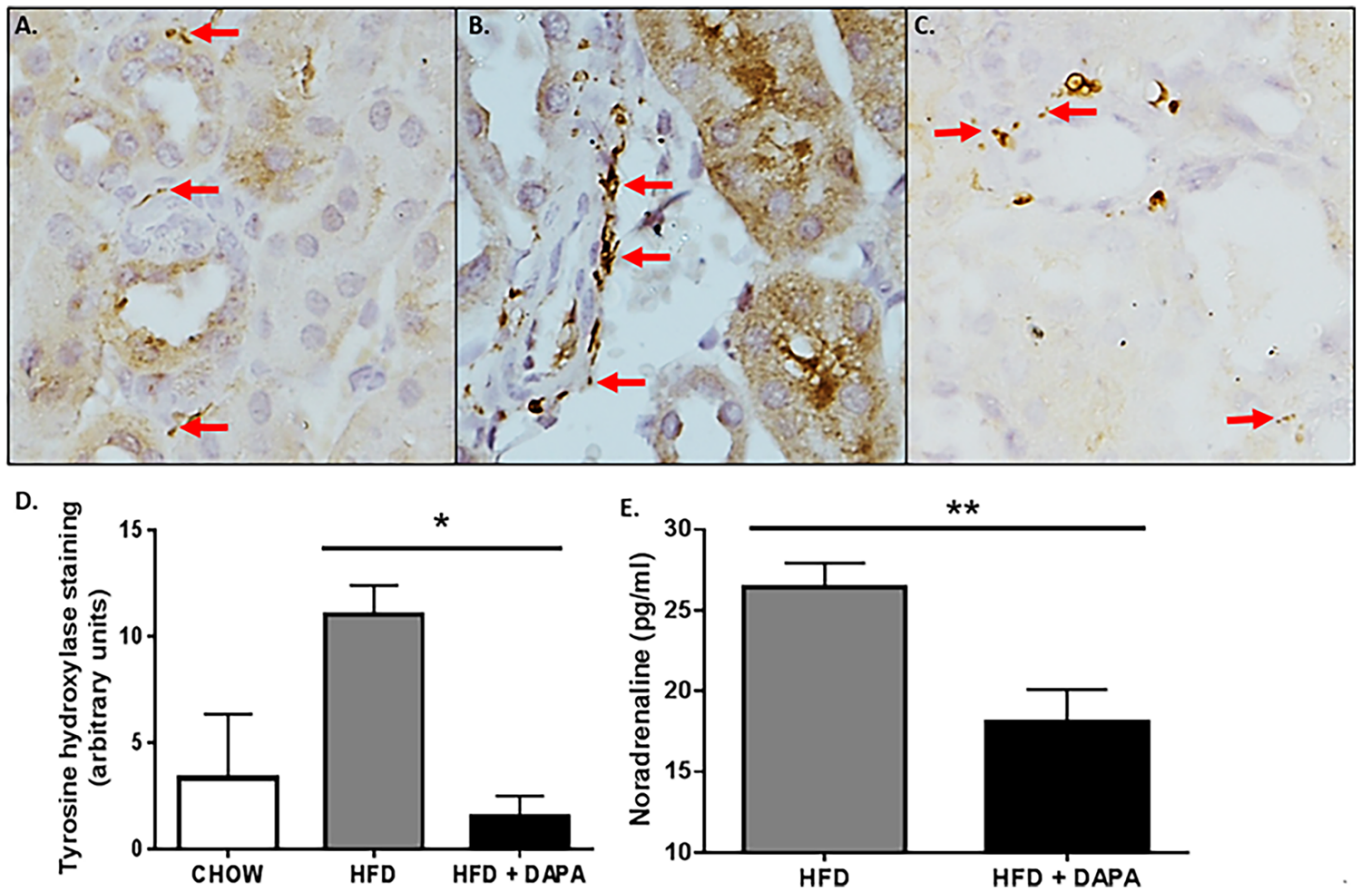
Tyrosine hydroxylase expression is reduced in kidney from mice fed a high-fat diet and dapagliflozin (DAPA). Representative immunohistochemistry images of tyrosine hydroxylase expression in kidney from mice fed chow (**A**), high fat diet (HFD) (**B**) or HFD + DAPA treatment (**C**). Tyrosine **hydroxylase** staining is indicated with arrows. Magnification 200 × . **D** Kidney from high-fat diet fed mice had significantly greater tyrosine hydroxylase compared with HFD + DAPA-treated mice, *n* = 3–5 mice per group; *p < 0.0012; mean + SEM. **E** Noradrenaline content in kidney from HFD and HFD + DAPA mice, *n* = 4–13 mice/group; **p < 0.05; mean + SEM [49]

In our high-fat diet (HFD)–fed murine studies (mice with glucose intolerance and obesity), DAPA-treated mice displayed reduced BP, experienced weight loss, possessed decreased hyperglycaemia and increased glucose tolerance. Furthermore, untreated HFD-fed mice displayed increased expression of tyrosine hydroxylase and noradrenaline in the kidney and the heart, which was indicative of increased SNS innervation and activation, respectively. Interestingly, DAPA treatment in HFD-fed mice diminished both renal tyrosine hydroxylase and noradrenaline levels in mice presenting with the MetS (Fig. 3). For the first time, we showed that SGLT2 inhibition with DAPA was imparting metabolic benefits in our mouse model of MetS via sympathoinhibition [49]. These findings strongly suggest that SGLT2 inhibition is associated with sympathoinhibition [49, 50].

**Fig. 4:**
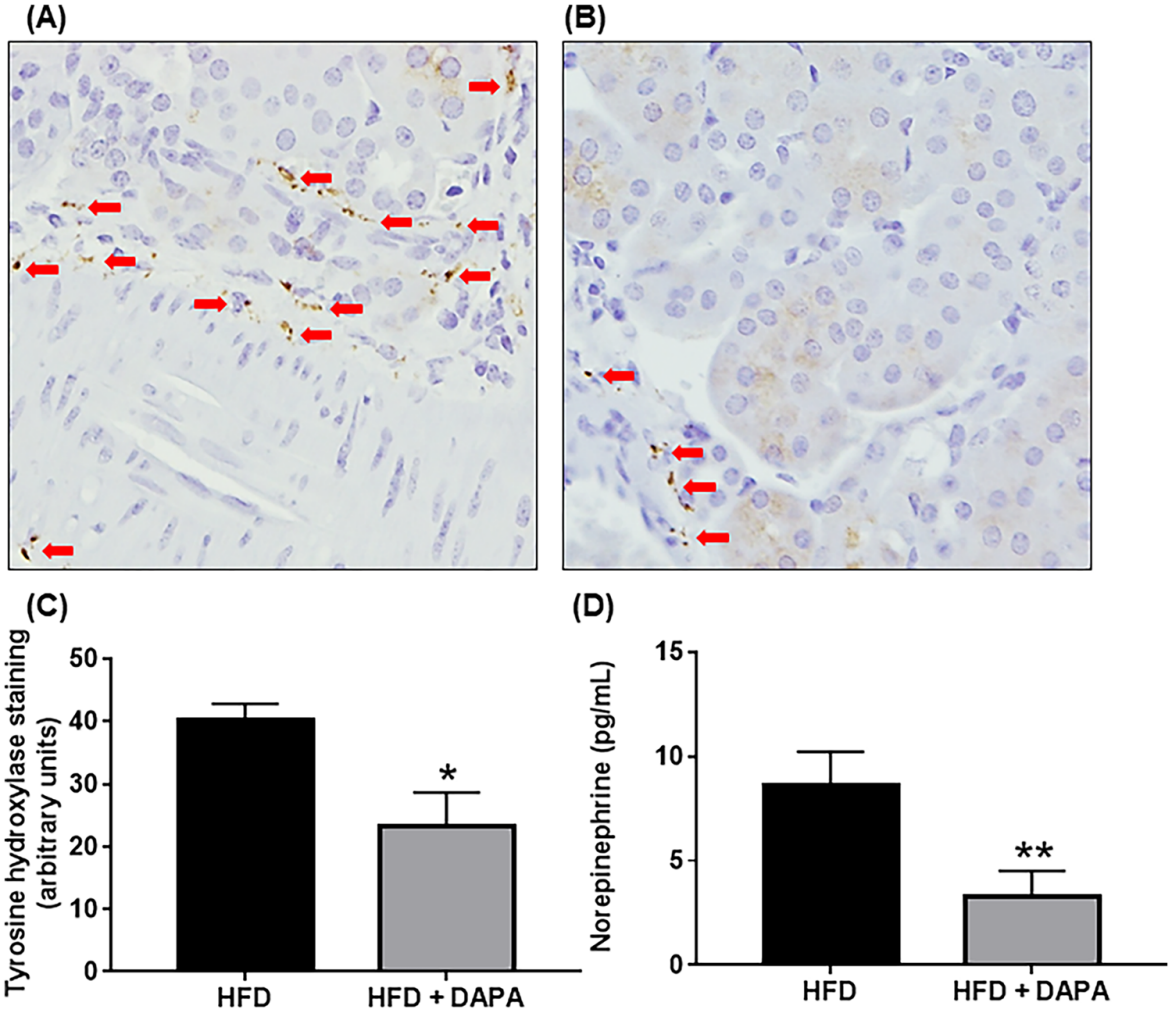
Inhibition of sodium glucose co-transporter 2 reduces activation of sympathetic nervous system in hypertensive mice. Representative immunohistochemistry images of tyrosine hydroxylase expression in kidney from mice fed a high-fat diet (HFD) (**A**) or HFD + DAPA treatment (**B**). Tyrosine hydroxylase staining is indicated with arrows. Magnification 200 × . **C** Tyrosine hydroxylase quantitation, *n* = 4–6 mice/group, *p = 0.04; mean ± SEM. **D** Norepinephrine content in kidney from HFD and HFD + DAPA mice, *n* = 15–19 mice/group; **p = 0.01; mean ± SEM [43••]

In our studies where the SNS has been downregulated via either chemical denervation or SGLT2 inhibition in the neurogenic hypertensive Schlager (BPH/2 J) mouse model, we have highlighted the following. Firstly, chemical denervation of the SNS promotes BP lowering, improved glucose homeostasis and decreased renal SGLT2 expression. Secondly, treatment with the SGLT2 inhibitor DAPA leads to significantly less weight gain, promotes BP lowering, protects against endothelial dysfunction, stimulates beneficial changes in the gut microbiome and decreases markers of SNS innervation and activity (Fig. 4). In conclusion, our innovative study highlights that sympathoinhibition with SGLT2 inhibitors promotes numerous metabolic benefits in the context of hypertension [43••].

It is evident that the regulation of adipose tissue is mediated by the SNS. The neurotransmitter noradrenaline is predominantly responsible for exerting fat metabolism via the SNS [51]. A recent murine study investigated the effect of CANA on HFD-induced obesity and its metabolic consequences. Treatment with CANA decreased fat mass and increased energy expenditure via increased thermogenesis and lipolysis in adipose tissue. Mechanistically, SGLT2 inhibition by CANA elevated adipose sympathetic innervation and fat mobilization via a β3-adrenoceptor-cAMP-PKA signalling pathway. Also, HFD fed mice treated with CANA showed improved insulin sensitivity and decreased hepatic steatosis. Taken together, it can be suggested that inhibition of SGLT2 increases energy consumption by increasing intra-adipose sympathetic innervation to counter diet-induced obesity and reveals a new therapeutic strategy by which SGLT2 inhibitors positively regulate energy homeostasis [52].

Excitingly, we report that although SGLT2 inhibition confers sympathoinhibition in many of the critical target organs, such as the heart and the kidneys [43••, 49], we have shown that DAPA may promote sympatho-excitation in white adipose tissue [53]. This leads to the beneficial phenomenon of beiging which was confirmed by elevated mRNA levels of the brown adipose tissue-selective gene *Ucp1* and the upstream mediator of *Ucp1*, *Pgc-1* [53]. It has been suggested that beiging of white adipose tissue enhances energy expenditure by reducing lipids stored within white adipose tissue. The phenomenon of beiging is considered a possible mechanism to counter obesity [54].

Also, in hypertensive atherosclerosis-prone mice (BPH/ApoE − / −), sympathetic activation accelerates the progression of atherosclerosis. In contrast, pharmacologically blocking sympathetic signalling resulted in decreased BP and atherosclerosis in these mice [55]. A recent study conducted in non-diabetic ApoE − / − mice highlighted that EMPA inhibited the progression of atherosclerosis by lipid lowering, reducing the inflammatory profile and downregulation of sympathetic activity as evidenced by decreases in markers of the SNS such as norepinephrine and neuropeptide Y [26••].

After consolidating these pre-clinical findings and concluding that the SGLT2 inhibition may result in sympathoinhibition, our group is now conducting a clinical trial to determine whether EMPA also exerts direct sympathoinhibitory effects on the heart and kidneys in human subjects with the MetS.

## Getting to the Heart of the Matter with SGLT2 Inhibition

Although SGLT2 inhibitors are effective glucose-lowering agents, the effectiveness of this drug class in patients with heart failure is unlikely related to improvements in glucose lowering per se [56]. Based on the available data summarized above, it appears likely that SGLT2 inhibitor-induced reduction in sympathetic activity may represent an important mediator of the beneficial effects of this drug class in heart failure.

The effect of SGLT2 inhibition on heart failure in patients with type 2 diabetes has been evaluated in several landmark clinical trials, including EMPA-REG (empagliflozin) [28], DELCARE-TIMI (dapagliflozin) [57••], CANVAS (canagliflozin) [30] and VERITAS CV (ertugliflozin; ERT) [58]. The widely used SGLT2 inhibitors have also been shown to greatly reduce the percentage of hospitalisations due to heart failure. For example, EMPA, DAPA, CANA and ERT have resulted in reductions of 35%, 27%, 33% and 30%, respectively [59]. To explain these findings, a critical mechanistic case report by Kiuchi et al. has highlighted that the SGLT2 inhibitor ipragliflozin, in a patient with chronic heart failure and diabetes mellitus, resulted in reduced cardiac sympathetic nerve hyperactivity. Of clinical importance, this patient was not re-hospitalized due to heart failure 2 years after administration of ipragliflozin started [60••].

Not all SGLT2 inhibitors are equally effective in reducing major adverse cardiac events (MACE). EMPA, DAPA and CANA had significant reductions in MACE (14%, 17% and 14%, respectively); however, ERT did not show a significant reduction. Recently, the DAPA-HF study was conducted to determine effects of SGLT2 inhibitors in patients with established heart failure and a reduced ejection fraction, regardless of the presence or absence of type 2 diabetes [61••]. In addition, DAPA was shown to reduce the risk of CV death and a first episode of worsening heart failure. The CV benefits imposed by SGLT2 inhibition in non-diabetics provide support for the notion that treatments such as SGLT2 inhibition have beneficial actions other than glucose lowering [61••].

## Conclusion

Our research team and others have demonstrated that SGLT2 inhibition is associated with a reduction in SNS activity, inhibition of norepinephrine turnover in brown adipose tissue and a reduction of tyrosine hydroxylase. These sympathoinhibitory effects appear to be observed in a diverse range of animal models, including models with and without diabetes/obesity [26••, 43••, 49, 55, 62, 63].

We are now focusing our research endeavours to also explore inhibition of the more widely expressed SGLT1 protein, as we have discovered that SGLT2 inhibition results in a compensatory increase in SGLT1 expression (unpublished data). The exciting novel dual inhibitor of SGLT1 and 2 (Sotagliflozin) has shown many metabolic benefits [64]. Therefore, further investigations involving dual SGLT1/2 inhibition during the MetS are warranted. It remains to be determined if dual inhibition results in more pronounced sympathoinhibition and thereby further improvements in BP and other metabolic parameters when compared to SGLT2 inhibition alone.
